# Inhibitory Synapse Formation at the Axon Initial Segment

**DOI:** 10.3389/fnmol.2019.00266

**Published:** 2019-11-05

**Authors:** Anna J. Nathanson, Paul A. Davies, Stephen J. Moss

**Affiliations:** ^1^Department of Neuroscience, Tufts University School of Medicine, Boston, MA, United States; ^2^AstraZeneca Tufts Laboratory for Basic and Translational Neuroscience, Boston, MA, United States; ^3^Department of Neuroscience, Physiology and Pharmacology, University College, London, United Kingdom

**Keywords:** GABA_A_ receptor, axon initial segment, collybistin, gephyrin, inhibition, synapse formation

## Abstract

The axon initial segment (AIS) is the site of action potential (AP) initiation in most neurons and is thus a critical site in the regulation of neuronal excitability. Normal function within the discrete AIS compartment requires intricate molecular machinery to ensure the proper concentration and organization of voltage-gated and ligand-gated ion channels; in humans, dysfunction at the AIS due to channel mutations is commonly associated with epileptic disorders. In this review, we will examine the molecular mechanisms underlying the formation of the only synapses found at the AIS: synapses containing γ-aminobutyric type A receptors (GABA_A_Rs). GABA_A_Rs are heteropentamers assembled from 19 possible subunits and are the primary mediators of fast synaptic inhibition in the brain. Although the total GABA_A_R population is incredibly heterogeneous, only one specific GABA_A_R subtype—the α2-containing receptor—is enriched at the AIS. These AIS synapses are innervated by GABAergic chandelier cells, and this inhibitory signaling is thought to contribute to the tight control of AP firing. Here, we will summarize the progress made in understanding the regulation of GABA_A_R synapse formation, concentrating on post-translational modifications of subunits and on interactions with intracellular proteins. We will then discuss subtype-specific synapse formation, with a focus on synapses found at the AIS, and how these synapses influence neuronal excitation.

## Introduction

The firing of glutamatergic pyramidal cells is tightly controlled by inhibitory interneurons (INs). By precisely directing pyramidal cell activity, INs are able to regulate network activity, generate oscillations, and even terminate pathological hyperexcitability (Fritschy, 2008; Roux and Buzsáki, 2015). On a molecular level, INs regulate pyramidal cell firing through GABAergic neurotransmission: releasing the neurotransmitter γ-aminobutyric acid (GABA) onto inhibitory postsynaptic specializations containing GABA type A receptors (GABA_A_Rs) on pyramidal neuron dendrites, soma, and axon initial segments (AISs). Thus, the construction and maintenance of GABAergic synapses are essential for normal inhibitory neurotransmission and brain function. However, relatively little is known about inhibitory synaptogenesis compared to glutamatergic synapses. To complicate the picture, there are many GABA_A_R subtypes composed of different subunits, which confer distinct physiological properties on the receptors. In addition, different GABA_A_R subtypes are selectively stabilized at different types of synapses; the AIS, for example, contains primarily one kind of GABA_A_R. Thus, the type of receptor present at a given synapse determines the type of inhibition that takes place. Again, little is known about how neurons direct different types of GABA_A_Rs to different synapses. The following review will briefly summarize what is known about the formation and trafficking of GABA_A_R subtypes and the construction of inhibitory synapses overall and specifically at the AIS.

## GABA_A_ Receptor Structure and Function

In the adult mammalian central nervous system, most fast, synaptic inhibitory neurotransmission is mediated by GABA_A_Rs, a group of heteropentameric, ligand-gated anion channels (Connolly and Wafford, 2004). When the neurotransmitter GABA binds to the receptor, the intrinsic ion pore opens and allows permeable ions to pass through (Bormann et al., 1987). GABA_A_Rs are primarily permeable to chloride (Cl^−^) anions (Fatima-Shad and Barry, 1993), and in the mature brain—where the Cl^−^ reversal potential is more negative than the resting membrane potential—the opening of the GABA_A_R channel allows Cl^−^ ions to flow down their electrochemical gradient into the neuron, lowering the neuron’s membrane potential and producing a hyperpolarizing response that reduces the probability of action potential (AP) firing (Busch and Sakmann, 1990; Blaesse et al., 2009).

Structurally, GABA_A_Rs are diverse. The receptors are assembled from 19 different known subunits: α(1–6), β(1–3), γ(1–3), δ, ε, θ, π, and ρ(1–3; Olsen and Sieghart, 2008), putting the number of possible subunit combinations in the thousands; however, only certain subtypes are expressed in the brain. For synaptic GABA_A_Rs, which this review will focus on, the typical stoichiometric ratio is as follows: 2α:2β:1γ (Wisden et al., 1992; Baumann et al., 2003). GABA_A_R subunits possess a similar amino acid sequence and protein structure, with each subunit composed of an extracellular N-terminal domain, four transmembrane domains (TM1–4), an intracellular loop domain (ICD) between TM3 and TM4, and an extracellular C-terminal domain (Schofield et al., 1987; Miller and Aricescu, 2014). The ICD is important for regulating GABA_A_R activity, as it is the site of phosphorylation and protein-protein interactions that alter receptor trafficking and plasma membrane (PM) expression (Moss et al., 1992; Nymann-Andersen et al., 2002; O’Toole and Jenkins, 2011). In addition, the ICD is the site of greatest sequence variability between subunits, making it an attractive candidate for a locus of subtype-specific GABA_A_R regulation (Arancibia-Cárcamo and Kittler, 2009). It seems likely that such differential regulation occurs, as different types of synaptic GABA_A_Rs are restricted to certain synapses. For instance, within pyramidal neurons in the cortex and hippocampus, GABA_A_Rs that contain the α1 subunit tend to be found at synapses in the soma and dendrites, while α2-containing GABA_A_Rs are enriched at synapses on the AIS (Nusser et al., 1996).

The subunit composition of a given GABA_A_R not only influences receptor localization, but also determines the physiological properties of that receptor (see Table 1 for summary). In addition, the specific α subunit composition of GABA_A_Rs determines receptor kinetics. α1-GABA_A_Rs mediate an inhibitory current with a longer decay time than α2-GABA_A_Rs (Goldstein et al., 2002). Thus, GABA_A_R subtypes mediate specific kinds of inhibition; restricting GABA_A_R subtypes to different spatial domains allows INs to control pyramidal neuron firing in a precise but dynamic manner.

**Table 1:** The distribution and synaptic roles of γ-aminobutyric acid type A receptor (GABA_A_R) α subunits.

Subunit	Brain distribution	Subcellular localization	Synaptic role
α1	60% of all GABA_A_Rs Widely expressed	Synaptic in somatodendritic compartments	Phasic inhibition
α2	15–20% of GABA_A_Rs Cerebral cortex (layers 1–4), hippocampus, striatum	Primarily synaptic; enriched in perisomatic regions and at the AIS of cortical and hippocampal pyramidal neurons	Phasic inhibition
α3	10%–15% of GABA_A_Rs Cerebral cortex (layers 5–6), amyddala, thalamus	Primarily synaptic; found in some AIS	Phasic inhibition
α4	<5% of GABA_A_R Dentate gyrus, thalamuss	Extrasynaptic	Tonic inhibition
α5	<5% of GABA_A_Rs Hippocampus	Extrasynaptic	Tonic inhibition
α6	<5% of GABA_A_Rs Cerebellum	Primarily extrasynaptic	Tonic inhibition

## GABA_A_ Receptor Oligomerization and Trafficking

GABA_A_R subunits are assembled into receptors in the endoplasmic reticulum (ER; Kittler et al., 2002). Oligomerization is controlled by the subunits’ N-terminal domains, with assistance from resident ER chaperone proteins to ensure appropriate protein assembly and folding (Connolly et al., 1996; Moss and Smart, 2001). Only those receptors that are conformationally mature are permitted to exit the ER and continue along the GABA_A_R lifecycle; receptors that are found to be incomplete or composed of inappropriate subunit combinations are retained in the ER and degraded (Gorrie et al., 1997; Saliba et al., 2007).

Conformationally mature GABA_A_Rs travel from the ER to the Golgi apparatus, where receptors are segregated into vesicles and transported to the PM (Vithlani et al., 2011). This forward trafficking delivers GABA_A_Rs to and insert them into the PM, primarily in extrasynaptic areas (Bogdanov et al., 2006). GABA_A_R surface expression is also regulated by receptor internalization *via* clathrin-mediated endocytosis (Lorenz-Guertin and Jacob, 2018). The clathrin adaptor protein (AP)-2 binds GABA_A_R subunits—the ICD of the GABA_A_R β1–3 and γ2 subunits both contain AP2 binding motifs—and clathrin, anchoring receptors in endocytotic pits.

## Inhibitory Synapse Construction

GABA_A_Rs are inserted into the PM at extrasynaptic locations (Bogdanov et al., 2006). At the surface, GABA_A_Rs are highly dynamic and diffuse laterally within the PM, where they continually move between the synaptic and extrasynaptic space (Thomas et al., 2005). Recent single-particle trafficking experiments show that both synaptic (α1–3-containing) and extrasynaptic (α4–6-containing) receptors can access the inhibitory synapse; however, when within the synaptic domain, the diffusion rate of synaptic GABA_A_Rs was reduced relative to extrasynaptic receptors, suggesting that GABA_A_Rs with “synaptic” subunit compositions are selectively stabilized at synapses (Hannan et al., 2019).

How are these receptors stabilized in the inhibitory synapse? Research to date suggests that protein-protein interactions play an essential role in this process: structural proteins present at the inhibitory synapse bind to GABA_A_Rs, reducing their lateral diffusion rate and effectively anchoring them at the synapse (Hannan et al., 2019). Though the composition of the multimolecular protein complexes present at the inhibitory synapse remains relatively unknown, a number of proteins that reside at the inhibitory synapse and appear to regulate GABA_A_R clustering have been identified.

## Gephyrin

One of the first inhibitory synaptic proteins described was gephyrin (GPN), which is still considered to be an integral structural component of the inhibitory postsynaptic domain (Tyagarajan and Fritschy, 2014). The most common splice variant of GPN is composed of three domains: an N-terminal G domain, a linker C domain, and a C-terminal E domain (Feng et al., 1998; Schwarz et al., 2001). The E and G domains of GPN self-aggregate, leading to the hypothesized formation of hexameric macromolecular GPN complexes that could serve as a lattice to stabilize receptors at the synapse (Saiyed et al., 2007). GPN was first identified as a binding partner of glycine receptors, which mediate inhibition in the spine (Prior et al., 1992). Constitutive knock-out of GPN in the mouse leads to a complete loss of glycine receptor clusters in the periphery, resulting in early postnatal death (Feng et al., 1998). However, it was also found that GPN knock-out mice show a dramatic reduction in the presence of GABA_A_Rs at brain synapses, providing the first evidence that GPN is also crucial for inhibitory synapse formation in the central nervous system (Kneussel et al., 1999; Fischer et al., 2000).

More recent experiments have shown that GPN co-localizes with GABA_A_Rs containing α1–3 subunits at synapses (Sassoè-Pognetto et al., 2000). Isothermal titration calorimetry experiments performed with the GPN E domain and the ICDs of GABA_A_R α1–3 subunits have demonstrated that GPN interacts directly with the ICD of GABA_A_R α subunits at an amino acid stretch between ICD residues 360–375 (Hines et al., 2018). The amino acid sequence in this region is not well conserved between α subunit subtypes, thus it follows that GPN binds α1–3 with differing affinities: the α1 and α3 ICDs formed tight complexes with the GPN E domain, while the α2 ICD formed a comparatively weaker complex (Hines et al., 2018). These data suggest a GABA_A_R subtype-specific affinity for GPN, dependent on the amino acid composition of the 360–375 ICD motif of the α subunit and raise the possibility that GPN, or other proteins that bind the 360–375 motif, can selectively stabilize GABA_A_R subtypes at certain synapses.

## Collybistin

A more recently identified inhibitory synapse protein is collybistin (CB), a guanine nucleotide exchange factor (Reid et al., 1999). Most functional CB isoforms are composed of three domains: a catalytic double homology domain, a PM-binding pleckstrin homology domain, and an N-terminal Src homology (SH)-3 domain (Harvey et al., 2004). CB was first identified as a GPN interacting protein (Kins et al., 2000). Indeed, the GPN E domain directly binds CB’s double homology domain, and co-expression of CB with GPN in heterologous cells causes the translocation of GPN clusters to the PM (Kins et al., 2000; Grosskreutz et al., 2001). CB knock-out mice show a loss of GPN clustering at inhibitory synapses in certain brain regions, such as the hippocampus, suggesting that CB plays a role in postsynaptic GPN clustering at a subset of inhibitory synapses (Papadopoulos et al., 2007, 2008).

Recent evidence showed that CB also directly interacts with certain GABA_A_R subtypes. Yeast tri-hybrid screens revealed that the GABA_A_R α2 subunit interacts with the CB SH3 domain, and in fact the GPN/CB interaction is strengthened by the addition of α2, suggesting that these three proteins can act synergistically (Saiepour et al., 2010). *In vitro* isothermal titration calorimetry showed that the CB SH3 domain preferentially binds the α2 ICD, over either the α1 or α3 ICD, at residues 360–375, suggesting that this ICD motif is integral to GABA_A_R subtype-specific protein-protein interactions (Hines et al., 2018). Supporting this hypothesis, knocking the α2 360–375 motif into the α1 subunit in mice leads to increased immunoprecipitation of endogenous CB with the chimeric α1 subunit (Nathanson et al., 2019). This same study also showed an increase in the pull-down of GPN with mutant α1, demonstrating a possible synergistic interaction between CB/GPN/α2 ICD that is overall strengthened when the interaction between two partner proteins is enhanced (Nathanson et al., 2019). The overarching question becomes: does this α2 ICD motif and its preferential protein interactions play a role in subtype-specific synapse formation in the brain, particularly in the construction of α2-enriched synapses at the AIS?

## The Axon Initial Segment

At the interface between the somatodendritic and axonal compartments lies the AIS. This discrete region is composed of unique molecular machinery and maintains a barrier between the somatodendritic and axonal environments, sustaining the neuronal anatomical asymmetry necessary for the unidirectional propagation of information (Leterrier, 2018). Morphologically, the AIS displays an electron-dense submembranous granular layer composed of a high density of voltage-gated ion channels and the highly organized, periodic protein scaffold that supports them (Xu et al., 2013). A number of electrophysiological studies established that the AIS is not only a barrier but is also the site of AP generation, as belied by its high resident concentrations of voltage-gated sodium and potassium channels, which are essential for the propagation of APs (Araki and Otani, 1955; Coombs et al., 1957; Fuortes et al., 1957).

Giant Ankyrin G is the key scaffolding protein and master organizer at the AIS; it recruits other essential AIS components, such as βIV-spectrin and voltage-gated ion channels, through either direct or indirect interactions (Zhou et al., 1998; Jenkins and Bennett, 2001; Han et al., 2017). Ankyrin G also interacts with microtubules, anchoring the entire complex in place (Leterrier et al., 2011). The AIS protein scaffold is dense and super-stable, maintaining axonal integrity and serving as a barrier to the entry of inappropriate somatodendritic proteins: the expression and/or stabilization of proteins at the AIS is tightly controlled (Albrecht et al., 2016; Huang and Rasband, 2016).

## Inhibition at the Axon Initial Segment

To current knowledge, the only ligand-gated ion channels mediating neurotransmission at the AIS are GABA_A_Rs (Leterrier, 2018). The AIS of certain cell types—pyramidal cells of the forebrain, for instance—contain inhibitory synapses that are exclusively innervated by one type of IN: the chandelier cell (Somogyi et al., 1983; Wang et al., 2016). Given that the AIS is the site of AP firing, any inhibitory signaling in this domain has an outsize effect on neuronal excitability (Zhu et al., 2004; Glickfeld et al., 2009). As previously discussed, α2-GABA_A_Rs are specifically enriched at the AIS (Nusser et al., 1996; Nyíri et al., 2001); since different GABA_A_R subtypes have their own kinetics and mediate distinct types of inhibition, it follows that the enrichment of a particular GABA_A_R subtype in a restricted domain like the AIS would have functional relevance.

To investigate the above hypothesis, mice in which residues 360–375 of the GABA_A_R α1 subunit have been knocked-in to the α2 subunit (*Gabra*2–1 mice) were generated. This mutation abolished α2’s preferential interaction with CB and led to loss of α2+ synapses at the AIS. Strikingly, *Gabra*2–1 animals display postnatal spontaneous seizures; these seizures are often lethal, causing death around postnatal day 20 (Hines et al., 2018). These data demonstrate that the localization of α2-GABA_A_Rs to the AIS is essential to inhibitory control of pathological excitation.

## Inhibitory Synapse Formation at the Axon Initial Segment

Clearly then, inhibition at the AIS is integral to maintaining the dynamic balance between inhibition and excitation. However, the manner in which GABA_A_R subtype-specific axo-axonic synapses are constructed and maintained remains unclear. Although α2-GABA_A_Rs are enriched at the AIS, live imaging of α1- and α2-GABA_A_Rs coupled to quantum dots showed that both subtypes can enter the AIS: the AIS diffusion barrier does not seem to select for α2-GABA_A_Rs (Muir and Kittler, 2014). However, these same studies demonstrated that α2-GABA_A_Rs were less mobile at the AIS than α1-GABA_A_Rs, indicating that while both subtypes can access the AIS compartment, α2-GABA_A_Rs are somehow preferentially anchored at there. Given that inhibitory synapse formation in other neuronal compartments has been shown to depend on protein-protein interactions, it stands to reason that synapse formation at the AIS would follow the same principles.

Indeed, GPN is expressed at the AIS, forming co-clusters with α2-GABA_A_Rs (Panzanelli et al., 2011), although GPN’s association with GABA_A_Rs at the AIS is relatively weaker than its association with GABA_A_Rs at the soma and dendrites (Gao and Heldt, 2016), suggesting that another protein present in the AIS multimolecular scaffold could play a more important role. CB is also present at AIS inhibitory synapses in cortical and hippocampal neurons (Panzanelli et al., 2011), and its specific interactions with α2-GABA_A_Rs provide a putative model for inhibitory synapse formation at the AIS: removing the 360–375 motif from the α2 subunit ICD prevents the accumulation of α2-GABA_A_Rs at axo-axonic synapses, suggesting that this motif, and the preferential protein interactions it mediates—such as that with CB—is *necessary* for GABA_A_R stabilization at the AIS (Hines et al., 2018). Experiments performed in another mutant mouse, in which residues 360–375 of the α2 subunit are knocked-in to the α1 subunit (the *Gabra*1–2 mouse), increases the affinity of the α1 subunit for CB and leads to an increase in α1-GABA_A_Rs expression at axo-axonic synapses. These data show that residues 360–375 of the α2 subunit are *sufficient* for GABA_A_R stabilization at the AIS (Nathanson et al., 2019). Given that CB has a relatively stronger association with the α2 360–375 motif and is present at the AIS, it stands to reason that CB interactions selectively stabilize α2-GABA_A_Rs at inhibitory AIS synapses.

**Figure 1:**
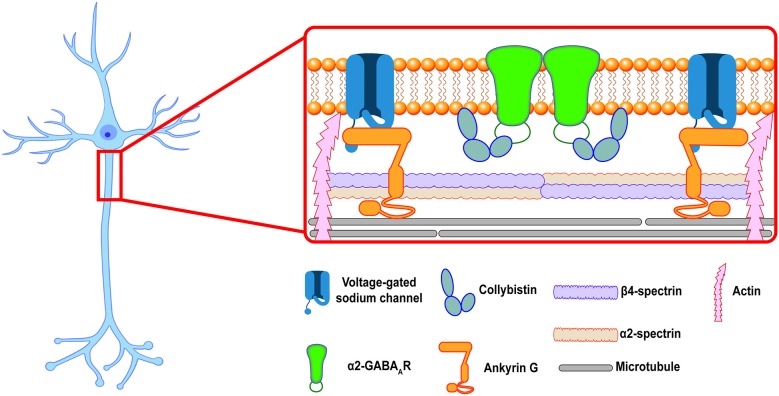
The inhibitory postsynaptic specialization at the axon initial segment (AIS). A cartoon showing a putative model of the postsynaptic inhibitory synapse at the AIS in a hippocampal pyramidal neuron. Ankyrin G and the β4/α2-spectrin tetramer associate to stabilize voltage-gated ion channels and link the periodic domain to the actin and microtubule cytoskeleton. α2-GABA_A_Rs are enriched at inhibitory synapses at the AIS, where they are selectively stabilized by protein-protein interactions at their intracellular loop domain (ICD). This review proposes that collybistin is a candidate for an AIS selective stabilizer, linking the α2-GABA_A_R to the AIS plasma membrane (PM).

Together, these data provide a potential model for axo-axonic synapse formation: after GABA_A_Rs are inserted into the extrasynaptic PM at the AIS those receptors that contain the α2 ICD motif are able to bind intracellular scaffolding proteins, such as CB, to form stable complexes that anchor the receptor at axo-axonic synapses. Receptors that do not contain the α2 ICD motif are not stabilized at synapses and diffuse back into the extrasynaptic space (see Figure 1). Other proteins in the AIS scaffold, especially Ankyrin G, might also play a role in the selective stabilization of GABA_A_Rs at the AIS. Future experiments utilizing the *Gabra*1–2 and *Gabra*2–1 mice could provide more information about the importance of these proteins in GABA_A_R stabilization. In addition, the above model only describes the *postsynaptic* side of inhibitory synapse formation. Additional mechanisms regulate the formation of presynaptic chandelier cell boutons apposing the AIS. Most recently, a transsynaptic mechanism was described: the cell adhesion molecule L1CAM, localized to the AIS of neocortical pyramidal neurons, was found to be necessary for the targeting of chandelier cell boutons to the AIS (Tai et al., 2019). Although the presynaptic interactor of L1CAM remains unidentified, such transsynaptic interactions provide an intriguing path for future research into synapse formation at the AIS.

## Conclusions

Despite the progress made in understanding the formation of inhibitory synapses, little is known about how neurons direct GABA_A_R subtype-specific synapse formation. This subtype specificity is important for the maintenance of neuronal excitability. α2-GABA_A_R-enriched synapse formation at the AIS is an especially intriguing case, as AIS inhibition is essential for normal brain function. Better understanding axo-axonic synapse formation will not only shed light on the molecular mechanisms of subtype-specific inhibitory synapse formation but may also provide new avenues of research into treatment for neurological disorders like epilepsy, which result from pathological hyperexcitability. It appears that protein-protein interactions between the ICD of GABA_A_R subunits and intracellular scaffolding proteins at inhibitory synapses play an important role in this process. The make-up of the inhibitory synaptic scaffold is variable depending on cell type and subcellular domain, making such interactions good candidates for synapse-specific GABA_A_R subtype enrichment. Further research will need to be done to fully explore the “interactome” of each GABA_A_R subtype and the importance of each interaction at the many different types of synapses present in even one neuron.

## Author Contributions

AN wrote the manuscript, with input from PD and SM.

## Conflict of Interest

SM serves as a consultant for AstraZeneca, Bain Capital, and Sage Therapeutics, relationships that are regulated by Tufts University. SM is also a shareholder of SAGE Therapeutics. The remaining authors declare that the research was conducted in the absence of any commercial or financial relationships that could be construed as a potential conflict of interest. The handling Editor declared a past co-authorship with one of the authors SM.

## References

[B1] Albrecht D. Winterflood C. M. Sadeghi M. Tschager T. Noé F. Ewers H. (2016). Nanoscopic compartmentalization of membrane protein motion at the axon initial segment. J. Cell Biol. 215, 37–46. 10.1083/jcb.201603108 27697928 PMC5057285

[B2] Araki T. Otani T. (1955). Response of single motoneurons to direct stimulation in toad’s spinal cord. J. Neurophysiol. 18, 472–485. 10.1152/jn.1955.18.5.472 13252436

[B3] Arancibia-Cárcamo I. L. Kittler J. T. (2009). Regulation of GABA_A_ receptor membrane trafficking and synaptic localization. Pharmacol. Ther. 123, 17–31. 10.1016/j.pharmthera.2009.03.012 19374920

[B4] Baumann S. W. Baur R. Sigel E. (2003). Individual properties of the two functional agonist sites in GABA_A_ receptors. J. Neurosci. 23, 11158–11166. 10.1523/JNEUROSCI.23-35-11158.2003 14657175 PMC6741049

[B5] Blaesse P. Airaksinen M. S. Rivera C. Kaila K. (2009). Cation-chloride cotransporters and neuronal function. Neuron 61, 820–838. 10.1016/j.neuron.2009.03.003 19323993

[B6] Bogdanov Y. Michels G. Armstrong-Gold C. Haydon P. G. Lindstrom J. Pangalos M.. (2006). Synaptic GABA_A_ receptors are directly recruited from their extrasynaptic counterparts. EMBO J. 25, 4381–4389. 10.1038/sj.emboj.7601309 16946701 PMC1570424

[B7] Bormann J. Hamill O. P. Sakmann B. (1987). Mechanism of anion permeation through channels gated by glycine and γ-aminobutyric acid in mouse cultured spinal neurones. J. Physiol. 385, 243–286. 10.1113/jphysiol.1987.sp016493 2443667 PMC1192346

[B8] Busch C. Sakmann B. (1990). Synaptic transmission in hippocampal neurons: numerical reconstruction of quantal IPSCs. Cold Spring Harb. Symp. Quant. Biol. 55, 69–80. 10.1101/sqb.1990.055.01.009 1966772

[B10] Connolly C. N. Krishek B. J. McDonald B. J. Smart T. G. Moss S. J. (1996). Assembly and cell surface expression of heteromeric and homomeric γ-aminobutyric acid type A receptors. J. Biol. Chem. 271, 89–96. 10.1074/jbc.271.1.89 8550630

[B9] Connolly C. N. Wafford K. A. (2004). The Cys-Loop superfamily of ligand-gated ion channels: the impact of receptor structure on function. Biochem. Soc. Trans. 32, 529–534. 10.1042/bst0320529 15157178

[B11] Coombs J. S. Curtis D. R. Eccles J. C. (1957). The generation of impulses in motoneurones. J. Physiol. 139, 232–249. 10.1113/jphysiol.1957.sp005888 13492210 PMC1358726

[B12] Fatima-Shad K. Barry P. H. (1993). Anion permeation in GABA- and glycine-gated channels of mammalian cultured hippocampal neurons. Proc. R. Soc. Lond. B Biol. Sci. 253, 69–75. 10.1098/rspb.1993.0083 7690484

[B13] Feng G. Tintrup H. Kirsch J. Nichol M. C. Kuhse J. Betz H.. (1998). Dual-requirement for gephyrin in glycine receptor clustering and molybdoenzyme activity. Science 282, 1321–1324. 10.1126/science.282.5392.1321 9812897

[B15] Fritschy J.-M. (2008). Epilepsy, E/I balance and GABA_A_ receptor plasticity. Front. Mol. Neurosci. 1:5. 10.3389/neuro.02.005.2008 18946538 PMC2525999

[B14] Fischer F. Kneussel M. Tintrup H. Haverkamp S. Rauen T. Betz H.. (2000). Reduced synaptic clustering of GABA and glycine receptors in the retina of the gephyrin null mutant mouse. J. Comp. Neurol. 427, 634–648. 10.1002/1096-9861(20001127)427:4<634::aid-cne10>3.0.co;2-x 11056469

[B16] Fuortes M. G. F. Frank K. Becker M. C. (1957). Steps in the production of motoneuron spikes. J. Gen. Physiol. 40, 735–752. 10.1085/jgp.40.5.735 13428986 PMC2147645

[B17] Gao Y. Heldt S. A. (2016). Enrichment of GABA_A_ receptor α-subunits on the axonal initial segment shows regional differences. Front. Cell. Neurosci. 10:39. 10.3389/fncel.2016.00039 26973458 PMC4771769

[B18] Glickfeld L. L. Roberts J. Somogyi P. Scanziani M. (2009). Interneurons hyperpolarize pyramidal cells along their entire somatodendritic axis. Nat. Neurosci. 12, 21–23. 10.1038/nn.2230 19029887 PMC3505023

[B19] Goldstein P. A. Elsen F. P. Ying S.-W. Ferguson C. Homanics G. E. Harrison N. L. (2002). Prolongation of hippocampal miniature inhibitory postsynaptic currents in mice lacking the GABA_A_ receptor α1 subunit. J. Neurophysiol. 88, 3208–3217. 10.1152/jn.00885.2001 12466441

[B20] Gorrie G. H. Vallis Y. Stephenson A. Whitfield J. Browning B. Smart T. G.. (1997). Assembly of GABA_A_ receptors composed of A1 and B2 subunits in both cultured neurons and fibroblasts. J. Neurosci. 17, 6587–6596. 10.1523/JNEUROSCI.17-17-06587.1997 9254671 PMC6573131

[B21] Grosskreutz Y. Hermann A. Kins S. Fuhrmann J. C. Betz H. Kneussel M. (2001). Identification of a gephyrin-binding motif in the GDP/GTP exchange factor collybistin. Biol. Chem. 382, 1455–1462. 10.1515/bc.2001.179 11727829

[B22] Han B. Zhou R. Xia C. Zhuang X. (2017). Structural organization of the actin-spectrin-based membrane skeleton in dendrites and soma of neurons. Proc. Natl. Acad. Sci. U S A 114, E6678–E6685. 10.1073/pnas.1705043114 28739933 PMC5559029

[B23] Hannan S. Minere M. Harris J. Izquierdo P. Thomas P. Tench B.. (2019). GABA_A_R isoform and subunit structural motifs determine synaptic and extrasynaptic receptor localisation. Neuropharmacology [Epub ahead of print]. 10.1016/j.neuropharm.2019.02.022 30794836

[B24] Harvey K. Duguid I. C. Alldred M. J. Beatty S. E. Ward H. Keep N. H.. (2004). The GDP-GTP exchange factor collybistin: an essential determinant of neuronal gephyrin clustering. J. Neurosci. 24, 5816–5826. 10.1523/JNEUROSCI.1184-04.2004 15215304 PMC6729214

[B26] Hines R. M. Maric H. M. Hines D. J. Modgil A. Panzanelli P. Nakamura Y.. (2018). Developmental seizures and mortality result from reducing GABA_A_ receptor A2-subunit interaction with collybistin. Nat. Commun. 9:3130. 10.1038/s41467-018-05481-1 30087324 PMC6081406

[B27] Huang Y.-M. Rasband M. N. (2016). Organization of the axon initial segment: actin like a fence. J. Cell Biol. 215, 9–11. 10.1083/jcb.201609084 27697921 PMC5057289

[B28] Jenkins S. M. Bennett V. (2001). Ankyrin-G coordinates assembly of the spectrin-based membrane skeleton, voltage-gated sodium channels, and L1 CAMs at purkinje neuron initial segments. J. Cell Biol. 155, 739–746. 10.1083/jcb.200109026 11724816 PMC2150881

[B29] Kins S. Betz H. Kirsch J. (2000). Collybistin, a newly identified brain-specific GEF, induces submembrane clustering of gephyrin. Nat. Neurosci. 3, 22–29. 10.1038/71096 10607391

[B32] Kittler J. T. McAinsh K. Moss S. J. (2002). Mechanisms of GABA_A_ receptor assembly and trafficking: implications for the modulation of inhibitory neurotransmission. Mol. Neurobiol. 26, 251–268. 10.1385/mn:26:2-3:251 12428759

[B35] Kneussel M. Brandstätter J. H. Laube B. Stahl S. Müller U. Betz H. (1999). Loss of postsynaptic GABA_A_ receptor clustering in gephyrin-deficient mice. J. Neurosci. 19, 9289–9297. 10.1523/JNEUROSCI.19-21-09289.1999 10531433 PMC6782938

[B37] Leterrier C. (2018). The axon initial segment: an updated viewpoint. J. Neurosci. 38, 2135–2145. 10.1523/JNEUROSCI.1922-17.2018 29378864 PMC6596274

[B36] Leterrier C. Vacher H. Fache M.-P. d’Angles S. A. Castets F. Autillo-Touati A.. (2011). End-binding proteins EB3 and EB1 link microtubules to ankyrin G in the axon initial segment. Proc. Natl. Acad. Sci. U S A 108, 8826–8831. 10.1073/pnas.1018671108 21551097 PMC3102358

[B38] Lorenz-Guertin J. M. Jacob T. C. (2018). GABA type a receptor trafficking and the architecture of synaptic inhibition. Dev. Neurobiol. 78, 238–270. 10.1002/dneu.22536 28901728 PMC6589839

[B39] Miller P. S. Aricescu A. R. (2014). Crystal structure of a human GABA_A_ receptor. Nature 512, 270–275. 10.1038/nature13293 24909990 PMC4167603

[B41] Moss S. J. Doherty C. A. Huganir R. L. (1992). Identification of the CAMP-dependent protein kinase and protein kinase C phosphorylation sites within the major intracellular domains of the β 1, γ 2S, and γ 2L subunits of the γ-aminobutyric acid type A receptor. J. Biol. Chem. 267, 14470–14476. 1321150

[B40] Moss S. J. Smart T. G. (2001). Constructing inhibitory synapses. Nat. Rev. Neurosci. 2, 240–250. 10.1038/35067500 11283747

[B42] Muir J. Kittler J. T. (2014). Plasticity of GABA_A_ receptor diffusion dynamics at the axon initial segment. Front. Cell. Neurosci. 8:151. 10.3389/fncel.2014.00151 24959118 PMC4051194

[B43] Nathanson A. J. Zhang Y. Smalley J. L. Ollerhead T. A. Rodriguez Santos M. A. Andrews P. M.. (2019). Identification of a core amino acid motif within the α subunit of GABA_A_Rs that promotes inhibitory synaptogenesis and resilience to seizures. Cell Rep. 28, 670.e8–681.e8. 10.1016/j.celrep.2019.06.014 31315046 PMC8283774

[B44] Nusser Z. Sieghart W. Benke D. Fritschy J. M. Somogyi P. (1996). Differential synaptic localization of two major γ-aminobutyric acid type A receptor α subunits on hippocampal pyramidal cells. Proc. Natl. Acad. Sci. U S A 93, 11939–11944. 10.1073/pnas.93.21.11939 8876241 PMC38162

[B45] Nyíri G. Freund T. F. Somogyi P. (2001). Input-dependent synaptic targeting of A2-subunit-containing GABA_A_ receptors in synapses of hippocampal pyramidal cells of the rat. Eur. J. Neurosci. 13, 428–442. 10.1046/j.1460-9568.2001.01407.x 11168550

[B46] Nymann-Andersen J. Sawyer G. W. Olsen R. W. (2002). Interaction between GABA_A_ receptor subunit intracellular loops: implications for higher order complex formation. J. Neurochem. 83, 1164–1171. 10.1046/j.1471-4159.2002.01222.x 12437587

[B47] O’Toole K. K. Jenkins A. (2011). Discrete M3–M4 intracellular loop subdomains control specific aspects of γ-aminobutyric acid type A receptor function. J. Biol. Chem. 286, 37990–37999. 10.1074/jbc.M111.258012 21903587 PMC3207432

[B48] Olsen R. W. Sieghart W. (2008). International union of pharmacology. LXX. Subtypes of γ-aminobutyric acid(A) receptors: classification on the basis of subunit composition, pharmacology, and function. Update. Pharmacol. Rev. 60, 243–260. 10.1124/pr.108.00505 18790874 PMC2847512

[B49] Panzanelli P. Gunn B. G. Schlatter M. C. Benke D. Tyagarajan S. K. Scheiffele P.. (2011). Distinct mechanisms regulate GABA_A_ receptor and gephyrin clustering at perisomatic and axo-axonic synapses on CA1 pyramidal cells. J. Physiol. 589, 4959–4980. 10.1113/jphysiol.2011.216028 21825022 PMC3224886

[B51] Papadopoulos T. Eulenburg V. Reddy-Alla S. Mansuy I. M. Li Y. Betz H. (2008). Collybistin is required for both the formation and maintenance of GABAergic postsynapses in the hippocampus. Mol. Cell. Neurosci. 39, 161–169. 10.1016/j.mcn.2008.06.006 18625319

[B50] Papadopoulos T. Korte M. Eulenburg V. Kubota H. Retiounskaia M. Harvey R. J.. (2007). Impaired GABAergic transmission and altered hippocampal synaptic plasticity in collybistin-deficient mice. EMBO J. 26, 3888–3899. 10.1038/sj.emboj.7601819 17690689 PMC1994120

[B52] Prior P. Schmitt B. Grenningloh G. Pribilla I. Multhaup G. Beyreuther K.. (1992). Primary structure and alternative splice variants of gephyrin, a putative glycine receptor-tubulin linker protein. Neuron 8, 1161–1170. 10.1016/0896-6273(92)90136-2 1319186

[B53] Reid T. Bathoorn A. Ahmadian M. R. Collard J. G. (1999). Identification and characterization of HPEM-2, a guanine nucleotide exchange factor specific for Cdc42. J. Biol. Chem. 274, 33587–33593. 10.1074/jbc.274.47.33587 10559246

[B54] Roux L. Buzsáki G. (2015). Tasks for inhibitory interneurons in intact brain circuits. Neuropharmacology 88, 10–23. 10.1016/j.neuropharm.2014.09.011 25239808 PMC4254329

[B55] Saiepour L. Fuchs C. Patrizi A. Sassoè-Pognetto M. Harvey R. J. Harvey K. (2010). Complex role of collybistin and gephyrin in GABA_A_ receptor clustering. J. Biol. Chem. 285, 29623–29631. 10.1074/jbc.m110.121368 20622020 PMC2937993

[B56] Saiyed T. Paarmann I. Schmitt B. Haeger S. Sola M. Schmalzing G.. (2007). Molecular basis of gephyrin clustering at inhibitory synapses: role of G- and E-domain interactions. J. Biol. Chem. 282, 5625–5632. 10.1074/jbc.m610290200 17182610

[B57] Saliba R. S. Michels G. Jacob T. C. Pangalos M. N. Moss S. J. (2007). Activity-dependent ubiquitination of GABA_A_ receptors regulates their accumulation at synaptic sites. J. Neurosci. 27, 13341–13351. 10.1523/JNEUROSCI.3277-07.2007 18045928 PMC6673389

[B58] Sassoè-Pognetto M. Panzanelli P. Sieghart W. Fritschy J. M. (2000). Colocalization of multiple GABA_A_ receptor subtypes with gephyrin at postsynaptic sites. J. Comp. Neurol. 420, 481–498. 10.1002/(sici)1096-9861(20000515)420:4<481::aid-cne6=3.0.co;2-5 10805922

[B59] Schofield P. R. Darlison M. G. Fujita N. Burt D. R. Stephenson F. A. Rodriguez H.. (1987). Sequence and functional expression of the GABA_A_ receptor shows a ligand-gated receptor super-family. Nature 328, 221–227. 10.1038/328221a0 3037384

[B60] Schwarz G. Schrader N. Mendel R. R. Hecht H. J. Schindelin H. (2001). Crystal structures of human gephyrin and plant Cnx1 G domains: comparative analysis and functional implications. J. Mol. Biol. 312, 405–418. 10.2210/pdb1eav/pdb 11554796

[B61] Somogyi P. Nunzi M. G. Gorio A. Smith A. D. (1983). A new type of specific interneuron in the monkey hippocampus forming synapses exclusively with the axon initial segments of pyramidal cells. Brain Res. 259, 137–142. 10.1016/0006-8993(83)91076-4 6824927

[B62] Tai Y. Gallo N. B. Wang M. Yu J.-R. Van Aelst L. (2019). Axo-axonic innervation of neocortical pyramidal neurons by GABAergic chandelier cells requires AnkyrinG-associated L1CAM. Neuron 102, 358.e9–372.e9. 10.1016/j.neuron.2019.02.009 30846310 PMC6525570

[B63] Thomas P. Mortensen M. Hosie A. M. Smart T. G. (2005). Dynamic mobility of functional GABA_A_ receptors at inhibitory synapses. Nat. Neurosci. 8, 889–897. 10.1038/nn1483 15951809

[B64] Tyagarajan S. K. Fritschy J.-M. (2014). Gephyrin: a master regulator of neuronal function? Nat. Rev. Neurosci. 15, 141–156. 10.1038/nrn3670 24552784

[B65] Vithlani M. Terunuma M. Moss S. J. (2011). The dynamic modulation of GABA_A_ receptor trafficking and its role in regulating the plasticity of inhibitory synapses. Physiol. Rev. 91, 1009–1022. 10.1152/physrev.00015.2010 21742794 PMC4382539

[B66] Wang Y. Zhang P. Wyskiel D. R. (2016). Chandelier cells in functional and dysfunctional neural circuits. Front. Neural Circuits 10:33. 10.3389/fncir.2016.00033 27199673 PMC4854894

[B67] Wisden W. Laurie D. J. Monyer H. Seeburg P. H. (1992). The distribution of 13 GABA_A_ receptor subunit MRNAs in the rat brain. I. Telencephalon, diencephalon, mesencephalon. J. Neurosci. 12, 1040–1062. 10.1523/JNEUROSCI.12-03-01040.1992 1312131 PMC6576059

[B68] Xu K. Zhong G. Zhuang X. (2013). Actin, spectrin, and associated proteins form a periodic cytoskeletal structure in axons. Science 339, 452–456. 10.1126/science.1232251 23239625 PMC3815867

[B69] Zhou D. Lambert S. Malen P. L. Carpenter S. Boland L. M. Bennett V. (1998). AnkyrinG is required for clustering of voltage-gated na channels at axon initial segments and for normal action potential firing. J. Cell Biol. 143, 1295–1304. 10.1083/jcb.143.5.1295 9832557 PMC2133082

[B70] Zhu Y. Stornetta R. L. Zhu J. J. (2004). Chandelier cells control excessive cortical excitation: characteristics of whisker-evoked synaptic responses of layer 2/3 nonpyramidal and pyramidal neurons. J. Neurosci. 24, 5101–5108. 10.1523/JNEUROSCI.0544-04.2004 15175379 PMC6729194

